# (*E*)-4-{[(Pyridin-4-yl­methyl­idene)amino]­meth­yl}benzoic acid

**DOI:** 10.1107/S1600536811056212

**Published:** 2012-01-07

**Authors:** Sun Hwa Han, Soon W. Lee

**Affiliations:** a Department of Chemistry (BK21), Sungkyunkwan University, Natural Science Campus, Suwon 440-746, Republic of Korea

## Abstract

The title mol­ecule, C_14_H_12_N_2_O_2_, exhibits a V-shaped conformation with a dihedral angle of 59.69 (3)° between the benzene and pyridine rings. In the crystal, O—H⋯N hydrogen bonds link the mol­ecules into zigzag chains along [010].

## Related literature

For *d*-block coordination polymers containing linking ligands related to the title mol­ecule, see: Hou *et al.* (2011 ▶); Jang & Lee (2010 ▶); Lee & Lee (2010 ▶); Kim & Lee (2008 ▶); Jung & Lee (2009 ▶). For *d*–*f* coordination polymers with pyrid­yl–carboxyl­ate linking ligands, see: Bo *et al.* (2010 ▶); Tang *et al.* (2011 ▶).
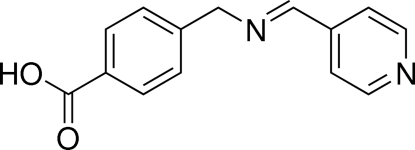


## Experimental

### 

#### Crystal data

C_14_H_12_N_2_O_2_
*M* *_r_* = 240.26
Monoclinic, 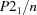

*a* = 4.2613 (1) Å
*b* = 26.5565 (6) Å
*c* = 10.3983 (2) Å
β = 98.123 (1)°
*V* = 1164.92 (4) Å^3^
*Z* = 4
Mo *K*α radiation
μ = 0.09 mm^−1^
*T* = 296 K
0.50 × 0.20 × 0.04 mm

#### Data collection

Bruker SMART CCD area-detector diffractometer
Absorption correction: multi-scan (*SADABS*; Sheldrick, 1996 ▶) *T*_min_ = 0.955, *T*_max_ = 0.996
22041 measured reflections
2899 independent reflections
1862 reflections with *I* > 2σ(*I*)
*R*_int_ = 0.067

#### Refinement

*R*[*F* ^2^ > 2σ(*F* ^2^)] = 0.047
*wR*(*F* ^2^) = 0.118
*S* = 1.03
2899 reflections
167 parameters
H atoms treated by a mixture of independent and constrained refinement
Δρ_max_ = 0.16 e Å^−3^
Δρ_min_ = −0.25 e Å^−3^

### 

Data collection: *SMART* (Bruker, 2007 ▶); cell refinement: *SAINT* (Bruker, 2007 ▶); data reduction: *SAINT*; program(s) used to solve structure: *SHELXTL* (Sheldrick, 2008 ▶); program(s) used to refine structure: *SHELXTL*; molecular graphics: *SHELXTL*; software used to prepare material for publication: *SHELXTL*.

## Supplementary Material

Crystal structure: contains datablock(s) I, global. DOI: 10.1107/S1600536811056212/cv5218sup1.cif

Structure factors: contains datablock(s) I. DOI: 10.1107/S1600536811056212/cv5218Isup2.hkl

Supplementary material file. DOI: 10.1107/S1600536811056212/cv5218Isup3.cml

Additional supplementary materials:  crystallographic information; 3D view; checkCIF report

## Figures and Tables

**Table 1:** Hydrogen-bond geometry (Å, °)

*D*—H⋯*A*	*D*—H	H⋯*A*	*D*⋯*A*	*D*—H⋯*A*
O1—H1*O*1⋯N1^i^	1.05 (2)	1.61 (2)	2.6634 (15)	178.7 (18)

Symmetry code: (i) 
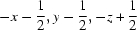
.

**Fig. 1.**
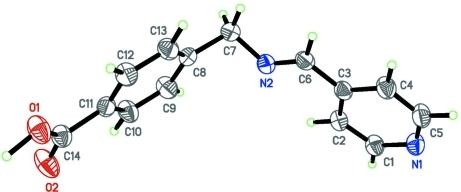
The molecular structure of the title compound showing the atomic numbering and 50% probabilty displacement ellipsoids.

**Fig. 2.**
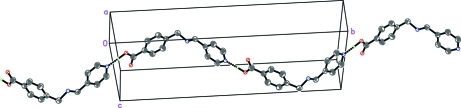
A portion of the crystal packing showing 1-D hydrogen-bonded (dashed lines) chain. C-bound H atoms omitted for clarity.

## supplementary crystallographic information

### Comment

There are many types of linking ligands containing various terminal groups, including pyridyl–pyridyl, pyridyl–amine, furan–furan, thiophene–thiophene, and pyridyl–carboxylate terminals. They are typically used to prepare coordination polymers (Hou *et al.*, 2011; Jang & Lee, 2010; Lee & Lee, 2010; Kim & Lee, 2008; Jung & Lee, 2009). In particular, pyridyl–carboxylate type linking ligands are unique in that they contain both an oxygen donor and a nitrogen donor. On the basis of the hard and soft acid–base theory, the harder oxygen atom is expected to coordinate to *f*-block metals and the softer nitrogen atom to *d*-block metals in *d*–*f* coordination polymers. Consistent with this expectation, such coordination modes have been observed in many *d*–*f* coordination polymers (Bo *et al.*, 2010; Tang, *et al.*, 2011). In our ongoing study of the preparation of coordination polymers, we obtained the title compound, which can be used as a linking ligand. Herewith we present its crystal structure.

The molecular structure of the title compound with the atom-labeling scheme is given in Figure 1, which clearly demonstrates both a pyridyl terminal and a carboxylate terminal in the title compound. The overall shape of the title compound can be described as V-shaped, with the dihedral angle of 59.69 (3)° between the phenyl ring (C8–C13) and pyridyl ring (N1, C1–C5). The carboxylate group (C14, O1, O2) is slightly twisted by 1.9 (2)° from the phenyl ring to which it is attached. As shown in Figure 2, molecules are connected by the strong intermolecular hydrogen bonds of the O–H···N type (Table 1). The H-bonds result in the formation of a one-dimensional zigzag chain along *b* axis.

### Experimental

4-(Aminomethyl)benzoic acid (0.38 g, 2.5 mmol) was added to 4-pyridinecarboxaldehyde (0.27 g, 2.5 mmol) in methanol (20 ml) at room temperature. The mixture was sealed in a 25 ml Teflon-lined vessel and heated at 73 °C for 18 h, and then slowly air-cooled. The resulting colorless crystals were filtered and then washed with methanol (10 ml × 3) to give the title compound (480 mg, 2.0 mmol, 80.0% yield). mp: 469–471 K. ^1^H NMR (500 MHz, CD_3_SOCD_3_, δ): 8.67–8.70 (d, 2H, pyridine N–C*H*), 8.57–8.60 (d, 2H, pyridine N–CC–*H*), 7.90–7.94 (d, 2H, aromatic protons), 7.33–7.36 (d, 2H, aromatic protons), 7.00 (m, 1H, N=C*H*), 4.87 (s, 2H, C*H*_2_). ^13^C{^1^H} NMR (125 MHz, CD_3_SOCD_3_, δ): 191.8, 170.6, 162.6, 149.3, 129.0, 128.6, 123.7, 122.6, 69.6, 64.2. IR (KBr, cm^-1^): 3047 (w), 2890 (w), 2837 (w), 2360 (w), 1926 (w), 1697 (m, CO), 1644 (w), 1607 (m, CN), 1564 (w), 1517 (w), 1448 (w), 1412 (w), 1384 (*m*), 1286 (*s*), 1170 (w), 1120 (w), 1091 (w), 1053 (w), 1017 (*m*), 986 (m\w), 951 (*m*), 825 (*m*), 766 (w), 699 (w), 655 (w).

### Refinement

C-bound H atoms were positioned geometrically [C—H = 0.93–0.97 Å], and allowed to ride on their parent atoms, with *U*_iso_(H) = 1.2 *U*_eq_(C). Atom H1O1 was located on a difference map and isotropically refined.

### Crystal data

**Table:**

C_14_H_12_N_2_O_2_	*F*(000) = 504
*M**_r_* = 240.26	*D*_x_ = 1.370 Mg m^−^^3^
Monoclinic, *P*2_1_/*n*	Mo *K*α radiation, λ = 0.71073 Å
Hall symbol: -P 2yn	Cell parameters from 6768 reflections
*a* = 4.2613 (1) Å	θ = 2.5–27.2°
*b* = 26.5565 (6) Å	µ = 0.09 mm^−^^1^
*c* = 10.3983 (2) Å	*T* = 296 K
β = 98.123 (1)°	Block, colourless
*V* = 1164.92 (4) Å^3^	0.50 × 0.20 × 0.04 mm
*Z* = 4	

### Data collection

**Table:**

Bruker SMART CCD area-detector diffractometer	2899 independent reflections
Radiation source: sealed tube	1862 reflections with *I* > 2σ(*I*)
graphite	*R*_int_ = 0.067
phi and ω scans	θ_max_ = 28.3°, θ_min_ = 1.5°
Absorption correction: multi-scan (*SADABS*; Sheldrick, 1996)	*h* = −5→5
*T*_min_ = 0.955, *T*_max_ = 0.996	*k* = −35→35
22041 measured reflections	*l* = −13→13

### Refinement

**Table:**

Refinement on *F*^2^	Primary atom site location: structure-invariant direct methods
Least-squares matrix: full	Secondary atom site location: difference Fourier map
*R*[*F*^2^ > 2σ(*F*^2^)] = 0.047	Hydrogen site location: inferred from neighbouring sites
*wR*(*F*^2^) = 0.118	H atoms treated by a mixture of independent and constrained refinement
*S* = 1.03	*w* = 1/[σ^2^(*F*_o_^2^) + (0.0608*P*)^2^ + 0.0198*P*] where *P* = (*F*_o_^2^ + 2*F*_c_^2^)/3
2899 reflections	(Δ/σ)_max_ < 0.001
167 parameters	Δρ_max_ = 0.16 e Å^−^^3^
0 restraints	Δρ_min_ = −0.25 e Å^−^^3^

### Special details

**Table:**

Geometry. All e.s.d.'s (except the e.s.d. in the dihedral angle between two l.s. planes) are estimated using the full covariance matrix. The cell e.s.d.'s are taken into account individually in the estimation of e.s.d.'s in distances, angles and torsion angles; correlations between e.s.d.'s in cell parameters are only used when they are defined by crystal symmetry. An approximate (isotropic) treatment of cell e.s.d.'s is used for estimating e.s.d.'s involving l.s. planes.
Refinement. Refinement of *F*^2^ against ALL reflections. The weighted *R*-factor *wR* and goodness of fit *S* are based on *F*^2^, conventional *R*-factors *R* are based on *F*, with *F* set to zero for negative *F*^2^. The threshold expression of *F*^2^ > σ(*F*^2^) is used only for calculating *R*-factors(gt) *etc*. and is not relevant to the choice of reflections for refinement. *R*-factors based on *F*^2^ are statistically about twice as large as those based on *F*, and *R*- factors based on ALL data will be even larger.

### Fractional atomic coordinates and isotropic or equivalent isotropic displacement parameters (Å^2^)

**Table:**

	*x*	*y*	*z*	*U*_iso_*/*U*_eq_	
O1	−0.0038 (3)	0.06332 (4)	0.16807 (10)	0.0586 (3)	
O2	0.0065 (3)	0.07470 (4)	0.38031 (10)	0.0686 (4)	
N1	−0.1682 (3)	0.48063 (4)	0.29341 (12)	0.0484 (3)	
N2	0.4831 (3)	0.32170 (4)	0.27822 (11)	0.0449 (3)	
C1	−0.0562 (3)	0.44698 (5)	0.38388 (13)	0.0469 (4)	
H1	−0.1024	0.4515	0.4679	0.056*	
C2	0.1236 (3)	0.40608 (5)	0.35942 (13)	0.0441 (3)	
H2	0.1961	0.3835	0.4254	0.053*	
C3	0.1953 (3)	0.39898 (5)	0.23455 (13)	0.0398 (3)	
C4	0.0795 (3)	0.43362 (5)	0.14082 (14)	0.0500 (4)	
H4	0.1227	0.4301	0.0561	0.060*	
C5	−0.0998 (4)	0.47320 (5)	0.17373 (14)	0.0537 (4)	
H5	−0.1776	0.4960	0.1092	0.064*	
C6	0.3881 (3)	0.35649 (5)	0.20028 (13)	0.0432 (3)	
H6	0.4426	0.3553	0.1168	0.052*	
C7	0.6761 (3)	0.28186 (5)	0.23166 (15)	0.0501 (4)	
H7A	0.8847	0.2818	0.2830	0.060*	
H7B	0.7018	0.2884	0.1420	0.060*	
C8	0.5224 (3)	0.23103 (5)	0.24140 (13)	0.0411 (3)	
C9	0.4602 (4)	0.21317 (6)	0.36002 (14)	0.0524 (4)	
H9	0.5154	0.2325	0.4343	0.063*	
C10	0.3177 (4)	0.16720 (5)	0.36952 (13)	0.0498 (4)	
H10	0.2795	0.1557	0.4504	0.060*	
C11	0.2303 (3)	0.13776 (5)	0.26076 (12)	0.0393 (3)	
C12	0.2926 (3)	0.15529 (5)	0.14231 (13)	0.0476 (4)	
H12	0.2361	0.1360	0.0679	0.057*	
C13	0.4390 (3)	0.20139 (5)	0.13340 (13)	0.0481 (4)	
H13	0.4819	0.2126	0.0529	0.058*	
C14	0.0674 (3)	0.08894 (5)	0.27622 (13)	0.0448 (3)	
H1O1	−0.134 (5)	0.0309 (7)	0.1844 (18)	0.103 (7)*	

### Atomic displacement parameters (Å^2^)

**Table:**

	*U*^11^	*U*^22^	*U*^33^	*U*^12^	*U*^13^	*U*^23^
O1	0.0841 (8)	0.0469 (6)	0.0460 (6)	−0.0180 (6)	0.0131 (5)	−0.0060 (5)
O2	0.1020 (9)	0.0593 (7)	0.0464 (6)	−0.0290 (6)	0.0169 (6)	0.0012 (5)
N1	0.0581 (7)	0.0364 (6)	0.0507 (7)	0.0011 (5)	0.0079 (6)	0.0011 (5)
N2	0.0482 (7)	0.0347 (6)	0.0532 (7)	−0.0032 (5)	0.0124 (6)	0.0004 (5)
C1	0.0596 (9)	0.0401 (8)	0.0416 (8)	−0.0019 (7)	0.0093 (7)	−0.0015 (6)
C2	0.0527 (8)	0.0375 (7)	0.0417 (8)	−0.0014 (6)	0.0051 (6)	0.0036 (6)
C3	0.0417 (7)	0.0333 (7)	0.0449 (8)	−0.0070 (6)	0.0073 (6)	−0.0001 (6)
C4	0.0644 (9)	0.0452 (8)	0.0419 (8)	0.0012 (7)	0.0131 (7)	0.0049 (6)
C5	0.0695 (10)	0.0432 (9)	0.0489 (9)	0.0063 (7)	0.0097 (8)	0.0092 (7)
C6	0.0449 (8)	0.0403 (8)	0.0461 (8)	−0.0058 (6)	0.0129 (6)	0.0008 (6)
C7	0.0470 (8)	0.0417 (8)	0.0649 (10)	0.0011 (6)	0.0189 (7)	0.0037 (7)
C8	0.0397 (7)	0.0352 (7)	0.0504 (8)	0.0051 (6)	0.0133 (6)	0.0014 (6)
C9	0.0672 (10)	0.0479 (8)	0.0437 (8)	−0.0111 (7)	0.0137 (7)	−0.0081 (7)
C10	0.0691 (10)	0.0459 (8)	0.0365 (7)	−0.0076 (7)	0.0151 (7)	0.0005 (6)
C11	0.0428 (7)	0.0362 (7)	0.0394 (7)	0.0032 (6)	0.0073 (6)	0.0001 (6)
C12	0.0599 (9)	0.0463 (8)	0.0373 (7)	−0.0027 (7)	0.0090 (7)	−0.0048 (6)
C13	0.0585 (9)	0.0475 (8)	0.0405 (8)	0.0010 (7)	0.0148 (7)	0.0074 (6)
C14	0.0521 (8)	0.0413 (8)	0.0412 (8)	−0.0016 (6)	0.0073 (6)	0.0003 (6)

### Geometric parameters (Å, °)

**Table:**

O1—C14	1.3127 (16)	C6—H6	0.9300
O1—H1O1	1.05 (2)	C7—C8	1.5101 (18)
O2—C14	1.2088 (15)	C7—H7A	0.9700
N1—C5	1.3325 (18)	C7—H7B	0.9700
N1—C1	1.3358 (17)	C8—C13	1.3764 (18)
N2—C6	1.2575 (16)	C8—C9	1.3817 (19)
N2—C7	1.4639 (17)	C9—C10	1.3734 (19)
C1—C2	1.3735 (19)	C9—H9	0.9300
C1—H1	0.9300	C10—C11	1.3819 (18)
C2—C3	1.3881 (18)	C10—H10	0.9300
C2—H2	0.9300	C11—C12	1.3774 (18)
C3—C4	1.3797 (18)	C11—C14	1.4899 (19)
C3—C6	1.4691 (18)	C12—C13	1.3828 (19)
C4—C5	1.371 (2)	C12—H12	0.9300
C4—H4	0.9300	C13—H13	0.9300
C5—H5	0.9300		
			
C14—O1—H1O1	110.4 (10)	N2—C7—H7B	109.5
C5—N1—C1	117.07 (12)	C8—C7—H7B	109.5
C6—N2—C7	117.54 (12)	H7A—C7—H7B	108.1
N1—C1—C2	123.49 (13)	C13—C8—C9	118.17 (12)
N1—C1—H1	118.3	C13—C8—C7	121.40 (12)
C2—C1—H1	118.3	C9—C8—C7	120.43 (13)
C1—C2—C3	118.88 (12)	C10—C9—C8	120.79 (13)
C1—C2—H2	120.6	C10—C9—H9	119.6
C3—C2—H2	120.6	C8—C9—H9	119.6
C4—C3—C2	117.78 (12)	C9—C10—C11	120.99 (13)
C4—C3—C6	119.81 (12)	C9—C10—H10	119.5
C2—C3—C6	122.40 (12)	C11—C10—H10	119.5
C5—C4—C3	119.42 (13)	C12—C11—C10	118.48 (12)
C5—C4—H4	120.3	C12—C11—C14	122.84 (12)
C3—C4—H4	120.3	C10—C11—C14	118.67 (12)
N1—C5—C4	123.37 (13)	C11—C12—C13	120.33 (13)
N1—C5—H5	118.3	C11—C12—H12	119.8
C4—C5—H5	118.3	C13—C12—H12	119.8
N2—C6—C3	123.23 (12)	C8—C13—C12	121.24 (12)
N2—C6—H6	118.4	C8—C13—H13	119.4
C3—C6—H6	118.4	C12—C13—H13	119.4
N2—C7—C8	110.77 (11)	O2—C14—O1	123.50 (13)
N2—C7—H7A	109.5	O2—C14—C11	122.08 (13)
C8—C7—H7A	109.5	O1—C14—C11	114.42 (12)

### Hydrogen-bond geometry (Å, °)

**Table:**

*D*—H···*A*	*D*—H	H···*A*	*D*···*A*	*D*—H···*A*
O1—H1O1···N1^i^	1.05 (2)	1.61 (2)	2.6634 (15)	178.7 (18)

Symmetry codes: (i) −*x*−1/2, *y*−1/2, −*z*+1/2.
